# HOMER2, a Stereociliary Scaffolding Protein, Is Essential for Normal Hearing in Humans and Mice

**DOI:** 10.1371/journal.pgen.1005137

**Published:** 2015-03-27

**Authors:** Hela Azaiez, Amanda R. Decker, Kevin T. Booth, Allen C. Simpson, A. Eliot Shearer, Patrick L. M. Huygen, Fengxiao Bu, Michael S. Hildebrand, Paul T. Ranum, Seiji B. Shibata, Ann Turner, Yuzhou Zhang, William J. Kimberling, Robert A. Cornell, Richard J. H. Smith

**Affiliations:** 1 Molecular Otolaryngology and Renal Research Laboratories, Department of Otolaryngology University of Iowa, Iowa City, Iowa, United States of America; 2 Department of Anatomy and Cell Biology, Carver College of Medicine, University of Iowa, Iowa City, Iowa, United States of America; 3 Department of Otorhinolaryngology, Radboud University Nijmegen Medical Centre, Nijmegen, Netherlands; 4 Austin Health, Department of Medicine, University of Melbourne, Melbourne, Australia; 5 Self-employed physician, Menlo Park, California, United States of America; 6 Interdepartmental PhD Program in Genetics, University of Iowa, Iowa City, Iowa, United States of America; Tel Aviv University, ISRAEL

## Abstract

Hereditary hearing loss is a clinically and genetically heterogeneous disorder. More than 80 genes have been implicated to date, and with the advent of targeted genomic enrichment and massively parallel sequencing (TGE+MPS) the rate of novel deafness-gene identification has accelerated. Here we report a family segregating post-lingual progressive autosomal dominant non-syndromic hearing loss (ADNSHL). After first excluding plausible variants in known deafness-causing genes using TGE+MPS, we completed whole exome sequencing in three hearing-impaired family members. Only a single variant, p.Arg185Pro in *HOMER2*, segregated with the hearing-loss phenotype in the extended family. This amino acid change alters a highly conserved residue in the coiled-coil domain of HOMER2 that is essential for protein multimerization and the HOMER2-CDC42 interaction. As a scaffolding protein, HOMER2 is involved in intracellular calcium homeostasis and cytoskeletal organization. Consistent with this function, we found robust expression in stereocilia of hair cells in the murine inner ear and observed that over-expression of mutant p.Pro185 *HOMER2* mRNA causes anatomical changes of the inner ear and neuromasts in zebrafish embryos. Furthermore, mouse mutants homozygous for the targeted deletion of *Homer2* present with early-onset rapidly progressive hearing loss. These data provide compelling evidence that HOMER2 is required for normal hearing and that its sequence alteration in humans leads to ADNSHL through a dominant-negative mode of action.

## Author Summary

The most frequent sensory disorder worldwide is hearing impairment. It impacts over 5% of the world population (360 million persons), and is characterized by extreme genetic heterogeneity. Over 80 genes have been implicated in isolated (also referred to as ‘non-syndromic’) hearing loss, and abundant evidence supports the existence of many more ‘deafness-causing’ genes. In this study, we used a sequential screening strategy to first exclude causal mutations in known deafness-causing genes in a family segregating autosomal dominant non-syndromic hearing loss. We next turned to whole exome sequencing and identified a single variant—p.Arg185Pro in *HOMER2*—that segregated with the phenotype in the extended family. To validate the pathological significance of this mutation, we studied two animal models. In zebrafish, we overexpressed mutant HOMER2 and observed inner ear defects; and in mice we documented robust expression in stereocilia of cochlear hair cells and demonstrated that its absence causes early-onset progressive deafness. Our data offer novel insights into gene pathways essential for normal auditory function and the maintenance of cochlear hair cells.

## Introduction

Targeted genomic enrichment and massively parallel sequencing (TGE+MPS) have revolutionized the field of hereditary hearing loss (HHL) by making comprehensive genetic testing for deafness a clinical reality and by accelerating the discovery of novel deafness-causing genes [1,2]. To date over 80 genes have been causally implicated in non-syndromic hearing loss (NSHL; Hereditary Hearing Loss Homepage). The proteins encoded by these genes are involved in a broad array of molecular and cellular mechanisms essential for the development and maintenance of normal auditory function [3]. At the centerpiece of this intricate system are the outer and inner hair cells (OHCs, IHCs)—key structures in the mechanotransduction process by which mechanical stimuli are translated into electrical impulses [4]. The precise and efficient tuning of OHCs and IHCs is closely linked to their anatomical integrity and the coordinated movement of their apical stereocilia.

The Homer proteins are scaffolding proteins crucial to many intracellular signaling cascades; their function underpins a variety of neuronal processes ranging from calcium homeostasis and cytoskeletal organization to synaptic plasticity associated with learning and memory [5,6]. Homer proteins are encoded by three genes; *HOMER1*, *2*, and *3* (MIM 604798, MIM 604799 and MIM 604800, respectively) that are translated into multiple isoforms as a result of alternative splicing [7]. All isoforms share an N-terminal conserved EVH1 (Ena VASP Homology 1) domain, which binds proline-rich regions of target proteins. The long isoforms (HOMER1b and c, HOMER2, HOMER3) additionally have a coiled-coil (CC) region and leucine zipper motifs in their divergent C-termini [8]. The CC region is required for homo/hetero-multimerization to form tetrameric hubs (in which the CC domains align in a parallel fashion) and for interaction with Rho family GTPase proteins like CDC42 (MIM 116952) [9–11]. Although the short isoforms lack CC domains and therefore do not form multimers, like their longer counterparts they bind target proteins through their EVH1 domain. This interaction regulates the activity of proteins involved in Ca^2+^ signaling complexes including metabotropic glutamate receptors (mGluR) [12], inositol tri-phosphate receptors (IP_3_R) [8] and transient receptor potential canonical channels (TRPC) [13]. Homer proteins also regulate basal cytosolic calcium via an interaction with the plasma membrane calcium reuptake pump, PMCA [14,15].

Aberrant Homer signaling has been associated with several developmentally related neurological syndromes including Fragile X syndrome, epilepsy, addiction, schizophrenia, neuropathic pain and Alzheimer’s disease [16–19]. To this list we now add autosomal dominant NSHL (ADNSHL).

## Results and Discussion

### Clinical description and progression of hearing loss

**Fig 1:**
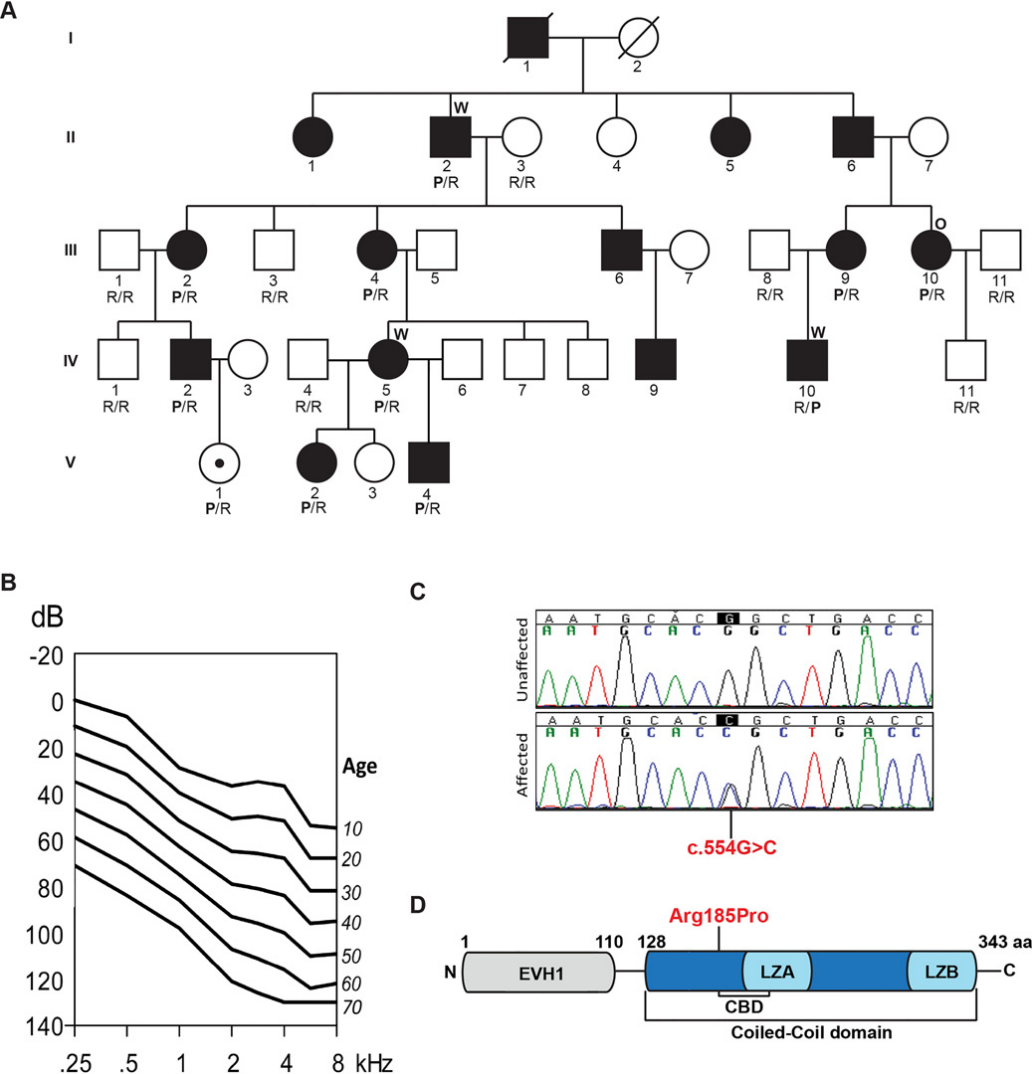
Pedigree, clinical data, HOMER2 mutation and protein structure. **(A)** The pedigree of a five-generation family segregating progressive ADNSHL. DNA samples were obtained for 9 unaffected and 10 affected individuals. The partially filled symbol represents a two-year-old female who carries the *HOMER2* mutation but in whom a formal ABR has not been performed. Individuals who underwent TGE+MPS using either the deafness panel (OtoSCOPE) or WES are marked with an O or W, respectively. Genotypes of participating family members are shown below each symbol in single letter amino acid nomenclature: P for Proline and R for Arginine. (**B)** Age-related typical audiograms (ARTA). Binaural mean air conduction thresholds (dB HL) are presented for the ages 10–70 years. Hearing levels ranged from 0 to 120 dB depending on age and frequency; the annual threshold deterioration (ATD) was 1.2–1.6 dB/year at all frequencies. (**C)** Representative chromatograms from wild-type and mutant sequences. (**D)** Diagram of HOMER2 structure; the amino acid numbering indicates the beginning and end of the EVH1 and CC domains. The CC includes the CDC42 binding domain (CBD), Leucine Zipper-A (LZA) and Leucine Zipper-B (LZB). The position of the p.Arg185Pro mutation is shown in red.

The family segregating the deafness-causing mutation in *HOMER2* (MIM 604799; RefSeq NM_004839) is a multi-generational kindred of European descent (Fig 1A). Pure tone audiometric evaluation of affected members showed bilateral post-lingual progressive hearing loss that segregated as an autosomal dominant trait; bone conduction thresholds excluded conductive hearing impairment. Clinical examination was negative for any findings consistent with syndromic hearing loss and also ruled out autoimmune phenotypes. Hearing impairment had a typical onset in the first decade of life in the high frequencies, with significant subsequent progression of hearing loss over all frequencies. To evaluate progression at each frequency, we performed linear regression analyses of threshold on age [20]. The resulting annual threshold deterioration (ATD) was 1.2 to 1.6 dB per year (Fig 1B, S1 Fig). The age-related typical audiogram (ARTA) derived from these data confirmed the down-sloping audiometric configuration and demonstrated fairly similar progression across all frequencies.

### Whole exome sequencing identifies a mutation in *HOMER2*

Our initial strategy was to screen one family member (III.10) for pathogenic variants in known deafness-causing genes using a deafness-specific TGE+MPS panel (OtoSCOPE) [21]. Plausible pathogenic variants were excluded (S1 Table). Whole exome sequencing (WES) was therefore completed on three affected family members (II.2, IV.5, and IV.10) and variants were filtered according to guidelines detailed in Materials and Methods section [22].

The resultant final candidate variant lists included 163, 150 and 158 nucleotide changes for these three individuals, respectively (S1 Table). Of these variants, only four were shared amongst the three sequenced exomes (S2 Table) and only one segregated with the ADNSHL phenotype in the extended family (Fig 1A and 1C). The segregating variant, c.554G>C; p.Arg185Pro in *HOMER2* on chromosome 15q25.2, is a novel non-synonymous change that substitutes a highly conserved arginine for a proline, a substitution that is predicted to be pathogenic and disease-causing by Polyphen2, LRT, SIFT and MutationTaster (S3 Table).

HOMER2 belongs to the homer family of post-synaptic density scaffolding proteins and is expressed as two isoforms, HOMER2 isoform 1 (NM_004839, 343 aa) and isoform 2 (NM_199330.2, 354 aa), which differ by 11 amino acids. The p.Arg185Pro variant lies in the CC domain (Fig 1D). A lysine is found at the orthologous position in HOMER1 and HOMER3, which like arginine is a basic polar amino acid that confers similar chemico-physical properties to the protein. Proline, in contrast, is non-polar and is predicted to alter the conformational structure of the CC domain or affect its ability to multimerize and/or interact with partner proteins.

### Homer2 localizes to stereocilia of hair cells in the murine cochlea

**Fig 2:**
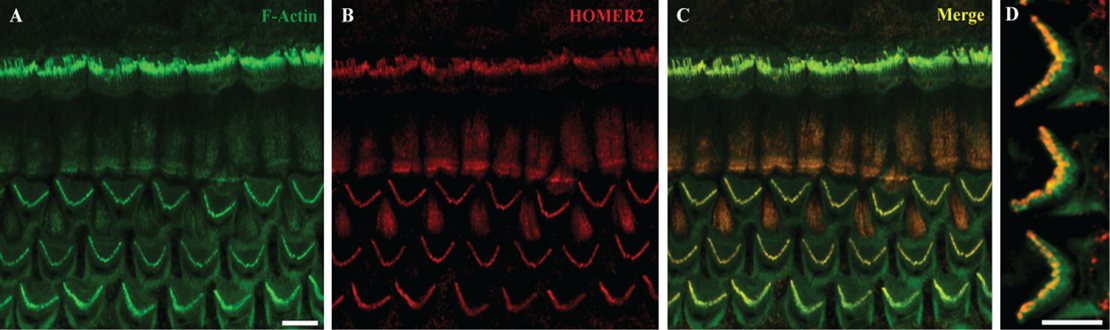
Homer2 expression in P2 mouse inner ear. **(A**) Staining with F-actin shows three rows of OHCs and one row of IHCs in the cochlea. (**B**) Homer2 staining in the OHCs and IHCs shows localization to stereocilia. (**C**) Merged pictures showing co-localization of Homer2 with F-actin in HC stereocilia. (**D**) Zoomed view of OHCs shows pronounced localization of Homer2 to the tips of stereocilia. Scale bar represents 10μm.

Homer2 is extensively expressed in the CNS throughout development [23]. It is also expressed in skeletal muscle, heart, liver, spleen, lung and kidney. To investigate and define its expression pattern in inner ear, we immuno-labeled whole mount P2 mouse cochlea with HOMER2 antibody. Organ of Corti expression was particularly enriched in the tips of stereocilia of both IHCs and OHCs (Fig 2), consistent with data by Hertzano and colleagues using cell sorting and RNASeq to identify Homer2 enrichment in the sensory cells of P0–P1 mice [24]. This expression pattern suggests a role for HOMER2 in hair bundle function, formation, development or maintenance.

### The p.Arg185Pro mutation does not affect subcellular localization in mammalian cells

To evaluate whether the p.Arg185Pro mutation affects subcellular localization, we transfected HEK293T and COS7 cells with cMYC-tagged HOMER2^WT^ proteins or FLAG-tagged HOMER2^p.Arg185Pro^. Both wild-type (WT) and mutant proteins distributed in a diffuse manner in the cytoplasm with no obvious differences in localization patterns (S2 Fig). A similar pattern of distribution was observed when both WT and mutant constructs were cotransfected. These data indicate that the p.Arg185Pro mutation does not alter subcellular localization of HOMER2.

### Overexpression of mutant HOMER2 in zebrafish embryos causes inner ear and neuromast defects

**Fig 3:**
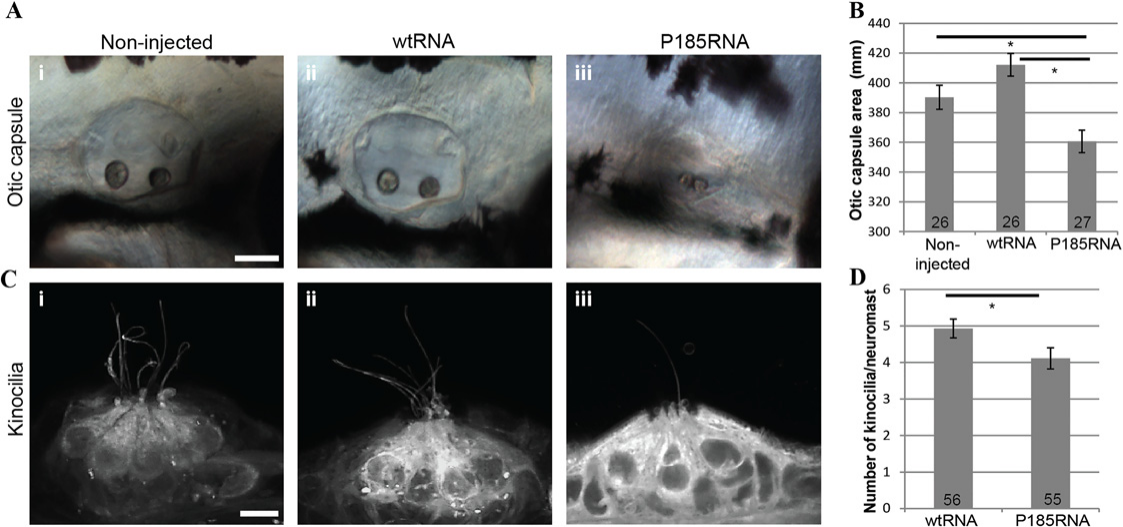
Over-expression of wtRNA and P185RNA in zebrafish embryos. **(A)** Otic capsule morphology in non-injected (i, n = 26), wtRNA-injected (ii, n = 26) and P185RNA-injected (iii, n = 27) embryos at 72 hpf. Scale bar represents 50μm. Embryos are mounted anterior to the left. (**B)** Comparison of otic capsule morphology between WT and mutant RNA injected zebrafish shows reduced inner ear size in mutant RNA injected larvae (p<0.001). **(C)** Hair cell kinocilia in P185RNA-injected larvae (iii) show fewer kinocilia as compared to non-injected (i) and wtRNA-injected larvae (ii). Scale bar represents 5μm. **(D)** Kinocilia count per neuromast comparison between wtRNA-injected (n = 56) and P185RNA-injected (n = 55). All data are presented as the mean ± SEM and are derived from at least triplicates (*P < 0.05 as compared with controls considered significant).

To assess the impact of the p.Arg185Pro mutation in HOMER2 *in vivo* we used the zebrafish model. Zebrafish hair cells share similarities with their mammalian counterparts in morphology and function. They reside inside the otic vesicles and in the neuromasts of the lateral line system, a sensory system on the surface of fish important for sensing propulsion through water, capturing prey, or avoiding predators and obstacles. The zebrafish *homer2* (NP_001018470.1) is 67% identical to human HOMER2 and is expressed mostly in the developing musculature although there is faint expression in the otic capsule at 24 hours post-fertilization (hpf) [25]. We used morpholino antisense oligonucleotides (S4 Table) to induce altered splicing and protein truncation. Although knockdown of *homer2* altered neither ear size nor morphology (p>0.5) (S3 Fig), injection of *in vitro* synthesized mRNA encoding HOMER2 P185-mutant RNA (P185RNA) resulted in significantly smaller ear size in larvae as compared to injections with WT HOMER2 (wtRNA) (p<0.001) (Fig 3A i-iii and 3B; S4 Fig). In addition, the number of kinocilia detected per neuromast was decreased (p = 0.03) (Fig 3C i-iii and 3D). We also noted that P185RNA-injected embryos exhibited shorter kinocilia, an observation that needs to be confirmed with more detailed methods. These results show that HOMER2 plays an essential role in the normal development and/or maintenance of hair cells in the zebrafish inner ear and that the p.Arg185Pro mutation has a dominant-negative effect on this process.

Dominant-negative activity has been previously validated *in vivo* for another homer protein, HOMER1. Its long isoforms, HOMER1b and 1c, are constitutively expressed while the short isoform, HOMER1a is an immediate early gene product that is rapidly and transiently induced by high synaptic activity [26]. Because HOMER1a lacks the CC domain, it cannot form multimers but it can still competitively bind the target proteins of HOMER1b and 1c, suggesting that relative expression of each of these homer proteins is critical [27–29]. The importance of this dynamic balance has been validated by the observation that overexpression of Homer1a in a mouse impedes normal development and through a dominant-negative effect leads to significant defects in motor coordination and learning, with increased levels of fear-associated behavior and anxiety [30].

### *Homer2* ^*-/-*^ mice exhibit early onset progressive hearing loss

**Fig 4:**
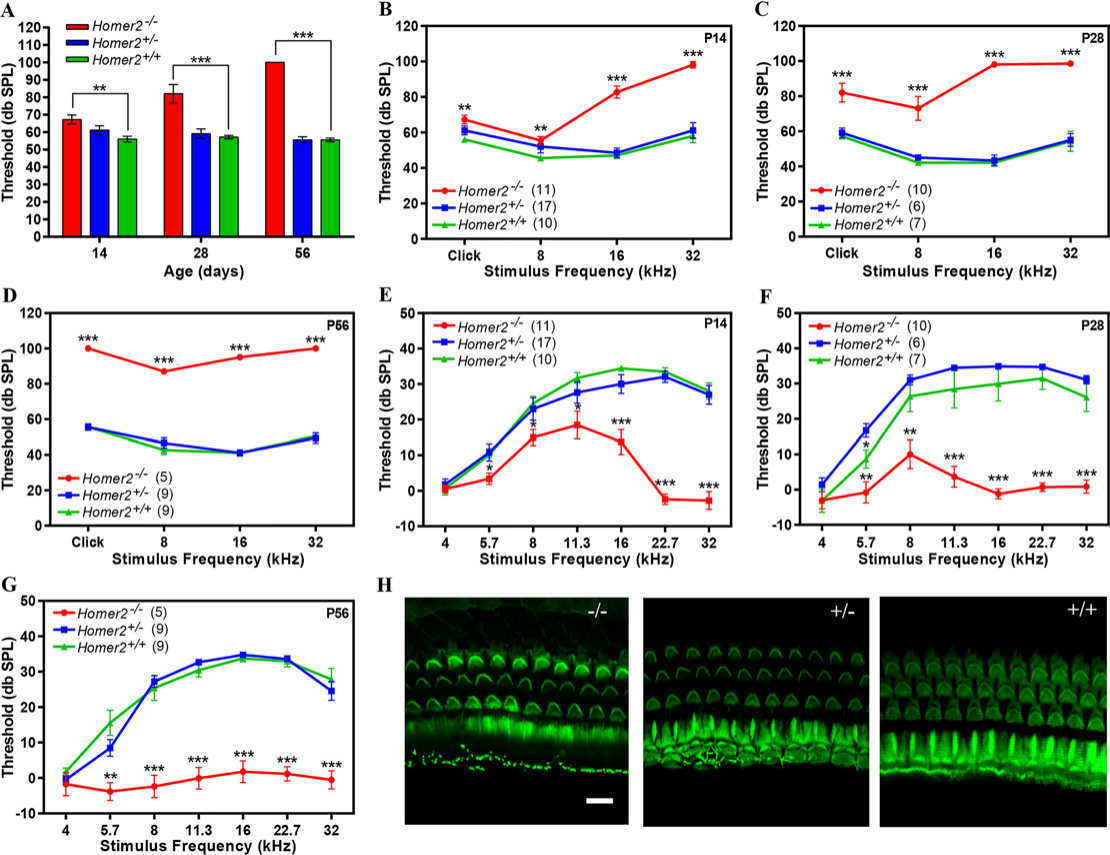
*Homer2* ^*-/-*^ mice have early onset progressive hearing loss. **(A-D)** Auditory brainstem responses (ABR) to broad band clicks (A) and tone bursts (B-D) at 8, 16, and 32 kHz in P14, P28 and P56 *Homer2* ^*-/-*^, *Homer2* ^*+/-*^ and WT mice. **(A)** Raised mean ABR thresholds (dB SPL) were detected as early as P14 in *Homer2* ^*-/-*^ mice and continued to increase with age. **(B)** At P14 *Homer2* ^*-/-*^ mice had severe-to-profound hearing loss at high frequencies (16 kHz and 32 kHz) whereas hearing thresholds in their WT and *Homer2* ^*+/-*^ littermates were in the normal range. **(C)** At P28 *Homer2* ^*-/-*^ mice had hearing loss in the lower frequencies. **(D)** P56 *Homer2* ^*-/-*^ mice had profound hearing loss across all tested frequencies (click, 8 kHz, 16 kHz and 32 kHz). **(E-G)** DPOAE levels. **(E)** In P14 *Homer2* ^*-/-*^ mice, DPOAE amplitudes are significantly lower in the high frequencies (16, 22.6, and 32.0 kHz) as compared to their WT and *Homer2* ^*+/-*^ littermates. **(F)** In P28 *Homer2* ^*-/-*^ mice, DPOAE amplitudes are lower in nearly all frequencies (5.7, 8, 11.3, 16, 22.6, and 32.0 kHz). **(G)** In P56 *Homer2* ^*-/-*^ mice, DPOAEs deteriorate across all frequencies consistent with profound hearing loss. **(H)** Representative Alexa-Fluor-488-phalloidin immunofluorescence shows no obvious hair cell death in cochleae in P56 *Homer2* ^*-/-*^ (n = 5), *Homer2* ^*+/-*^ (n = 4) and WT (n = 3) mice. Mean ABR thresholds and DPOAE amplitudes: *Homer2* ^*-/-*^ mice are shown in red; *Homer2* ^*+/-*^ in blue; and WT mice in green. The number of ears tested is shown in parentheses. P-values were calculated with One-way ANOVA and post-hoc T-test analysis. Asterisks indicate statistical significance: *P<0.05, **P<0.005, ***P<0.0005. Error bars represent SEM. Scale bar represents 10μm.

The role of Homer2 has been thoroughly studied in the murine brain and pancreas where it functions to decrease the intensity of Ca^2+^ signaling by reducing signaling by G protein-coupled receptors (GPCRs) [31]. Mice homozygous for the targeted deletion of homer2 (*Homer2* ^*-/-*^ mice generated by deletion of exon 3 [32]) show extensive behavioral and neurochemical similarities to cocaine or alcohol-sensitized animals, and demonstrate Homer2 involvement in appetitive pathways underlying responses to those drugs and their induced behavioral/cellular neuroplasticity within the nucleus accumbens [33,34]. Studies in *Homer2* ^*-/-*^ *Homer3* ^*-/-*^ mice show upregulation of cytokine expression and an increase in effector-memory T cells leading to an autoimmune-like pathology, indicating that Homer2 negatively regulates T cell activation [35]. To evaluate cochlear function of *Homer2* ^*-/-*^ mice, we measured auditory brainstem responses (ABR), an electrophysiological hearing test that reflects the activity of afferent auditory neurons downstream of IHCs. We examined hearing in 2-, 4- and 8-week-old *Homer2* ^*-/-*^, *Homer2* ^*+/-*^ and WT *Homer2* ^*+/+*^ mice and observed no differences between age-matched WT and *Homer2* ^*+/-*^ animals (P>0.2), however *Homer2* ^*-/-*^ mice showed progressive hearing loss (Fig 4). At P14 *Homer2* ^*-/-*^ mice had slightly elevated broad band click ABR thresholds as compared to *Homer2* ^*+/-*^ mice [*Homer2* ^*-/-*^ (n = 11); 67.3±2.56 verse *Homer2* ^*+/-*^ (n = 17); 61.20±2.50 db SPL]. This difference progressively increased ([P28 *Homer2* ^*-/-*^ (n = 10); 82.0±5.40 db SPL vs *Homer2* ^*+/-*^ (n = 6); 59.20±2.71 db SPL]; [P56 *Homer2* ^*-/-*^ (n = 5); 100.0±0.0 db SPL vs *Homer2* ^*+/-*^ (n = 9); 55.6±1.76 db SPL]) (Fig 4A). Tone bursts showed that P14 *Homer2* ^*-/-*^ mice had profound deafness at 32 kHz and that the rate of hearing deterioration at 8 kHz was dramatic (P14, 55.5±2.28 dbSPL; P28, 73.0±6.84; P56, 87.0±1.22 dbSPL) (Fig 4B-4D). We assessed OHC function using distortion product otoacoustic emissions (DPOAEs) and observed no significant differences in DPOAE thresholds between *Homer2* ^*+/-*^ and WT mice (P>0.05). In *Homer2* ^*-/-*^ mice, significant decreases in mid- to high-frequency DPOAE levels were seen at P14 and P28 that culminated in profound deafness at all frequencies by P56 (Fig 4E-4G).

To determine whether the auditory deficit in *Homer2* ^*-/-*^ mice was secondary to hair cell loss we analyzed whole mount preparations of the organ of Corti at P56. No differences in IHCs and OHCs were observed in any animals regardless of genotype indicating the absence of Homer2 does not impair hair cell formation and development, and that the hearing loss in *Homer2* ^*-/-*^ mice is not due to hair cell death (Fig 4H). Whether the hearing deficit in these mice is due to abnormal hair bundle morphology remains to be elucidated. We additionally investigated spiral ganglion morphology and found no obvious indication of spiral ganglion degeneration in *Homer2* ^*-/-*^ mice as compared to their WT littermates (S5 Fig).

### p.Arg185Pro mutation exerts its effect through a dominant-negative mechanism

In aggregate, our data show that HOMER2 is required for normal hearing. Its complete absence in mice leads to early onset progressive hearing loss starting at the high frequencies and rapidly involving all frequencies. The recessive phenotype exhibited by null alleles of Homer2 makes it a strong candidate for autosomal recessive hearing loss due to loss of function in humans as well. The absence of a disease phenotype in *Homer2* ^*+/-*^ mice suggests that haploinsufficiency does not cause hearing loss. Together with results obtained from zebrafish experiments, these data strongly suggest that the p.Arg185Pro mutation in HOMER2 exerts its effect through a dominant-negative mechanism on wild-type protein by either inhibiting multimerization or competing for other partner proteins.

In defining the effect of the p.Arg185Pro mutation in HOMER2 at a molecular level, we believe two hypotheses warrant consideration. One hypothesis posits that HOMER2 exerts its function by regulating actin dynamics in stereocilia through its interaction with CDC42, a highly conserved small GTPase of the RHO family that fine-tunes actin-turnover (S6 Fig). HOMER2 is known to couple with and regulate CDC42 through its CDC42-binding domain (CBD) within the CC domain (Fig 1D) [10]. In HeLa cells, CDC42 induces the formation of filopodia-like protrusions, while overexpression of HOMER2 suppresses this phenotype [36]. In the cochlea, CDC42 localizes to stereocilia membranes and its targeted deletion in murine HCs leads to their degeneration and results in progressive hearing loss particularly at the high frequencies, a phenotype similar to the human *HOMER2* mutant phenotype and the *Homer2* ^*-/-*^ murine phenotype [36].

A second hypothesis focuses on the role of HOMER2 in cytoplasmic Ca^2+^ control. Several studies have shown that HOMER2 regulates a number of Ca^2+^ handling proteins including TRPC and PMCA channels. Two TRPCs—TRPC3 (MIM 602345) and TRPC6 (MIM 603652)—are expressed in both sensory neurons and cochlear hair cells and are required for normal function. Their targeted deletion in mice causes significant dysregulation of Ca^2+^ re-entry that leads to hearing impairment and vestibular deficits [37]. While both proteins are potentially interacting partners for HOMER2, to date only an interaction with TRPC1 (MIM 602343) has been established [13]. However, a recent study has demonstrated a critical role for Homer2 in modulating PMCA activity by regulating the duration of the Ca^2+^ signaling in parotid acinar cells [38]. This finding suggests a possible role for Homer2 in cytosolic Ca^2+^ clearance to balance TRPC-mediated Ca^2+^ influx and PMCA-mediated Ca^2+^ extrusion. A suitable interacting partner of HOMER2 may be the PMCA2 pump (MIM 108733), which represents the only system for clearance of Ca^2+^ from hair cell stereocilia [39]. While both of these hypotheses are attractive, further functional studies are needed to identify the partner proteins of HOMER2 in inner ear and investigate the effect of the p.Arg185Pro mutation on these interactions.

In summary, we have identified *HOMER2* as essential to normal auditory function and have shown that the p.Arg185Pro HOMER2 mutation causes ADNSHL through a dominant-negative mechanism of action, thus expanding the phenotypic spectrum associated with Homer protein dysfunction.

## Materials and Methods

### Subjects and ethics statement

A five-generation family of European descent segregating bilateral post-lingual progressive ADNSHL was ascertained for this study (Fig 1A). After obtaining written informed consent from all participants with approval by the Institutional Review Board of the University of Iowa, clinical examination of the subjects was completed to exclude any additional and/or syndromic findings. Blood samples were obtained from 19 family members.

### Audiograms and data analysis

Pure tone audiometry was performed according to current standards to determine air conduction thresholds at 0.25, 0.5, 1, 2, 3, 4, 6 and 8 kHz. Bone conduction thresholds were determined at 0.5, 1, 2 and 4 kHz in some patient to exclude conductive hearing impairment. After validating binaural symmetry, the binaural mean air conduction threshold (dB Hearing Level, HL) at each frequency was used for further analyses. An arbitrary value of 130 dB HL was used to indicate out-of-scale measurements. Linear regression analyses of threshold on age were used to evaluate progression of hearing impairment at individual frequencies. These analyses comprised both individual longitudinal data derived from serial audiograms and overall cross-sectional last-visit data. Progression was considered significant if the 95% confidence interval for slope did not include zero for two or more frequencies. Progression was expressed in dB-per-year and designated Annual Threshold Deterioration (ATD). Cross-sectional regression data conformed to individual longitudinal regression data. Regression data from the last-visit thresholds were used to derive Age-Related Typical Audiograms (ARTA), which show expected thresholds by decade steps in age [20].

### Targeted genomic capture and whole exome sequencing

OtoSCOPE v1 was used to evaluate all known genetic causes of NSHL (including the non-syndromic mimics like Usher Syndrome) in one affected individual (III.10), as previously described [21,40]. Whole exome capture was performed with the Agilent SureSelectXT Human All Exon V4 (Agilent Technologies, Santa Clara, CA) according to the manufacturer’s protocol. All enriched libraries were sequenced on the Illumina HiSeq 2000 (Illumina, Inc., San Diego, CA) using 100bp paired-end reads. Data analysis was performed on a local installation of Galaxy using the Burrows-Wheeler Alignment (BWA) for read mapping to the reference genome (hg19, NCBI Build 37), Picard for removal of duplicate reads, GATK for local re-alignment and variant calling, and ANNOVAR and a custom workflow for variant annotation. Variant filtering was based on: quality (>10X); minor allele frequency (MAF<0.0005) as reported in the 1000 Genomes Project database and the National Heart, Lung, and Blood Institute (NHLBI) Exome Sequencing Project Exome Variant Server (EVS). Variants were annotated for conservation (GERP and PhyloP) and predicted pathogenicity (PolyPhen2, SIFT, MutationTaster and LRT). Variants were then filtered based on coding effect (non-synonymous, indels and splice-site variants); heterozygosity and allele sharing amongst the three sequenced affected individuals (II.2, IV.5, and IV.10). Sanger sequencing was completed in all family members to confirm segregation of c.554G>C; p.Arg185Pro in *HOMER2* gene (MIM 604799; RefSeq NM_004839) using primers HOMER2-6F: 5’-ATGGGAGAGGCAGCAAGTCT-3’ and HOMER2-6R: 5’-AGACCCACCTGCCAGCTTAC-3’.

### Immunostaining

Cochleae from Balb/c mice were harvested at P2, locally perfused, fixed in 4% paraformaldehyde for 30min, and rinsed in PBS. Tissues were microdissected into cochlear and saccule subsets and stored at 4°C in preparation for immunohistochemistry. Following infiltration using 0.3% Triton X-100 and blocking with 5% normal goat serum, we incubated the tissues in rabbit HOMER2 polyclonal primary antibody (NB100-98712, Novus Biologicals, Littleton, CO) diluted 1:1000 in PBS overnight at 4°C. Specificity of HOMER2 antibody was confirmed by staining whole mount cochlea from *Homer2* ^*-/-*^ mice (S7 Fig). Subsequently, a secondary antibody Alexa-Fluor 568 Goat anti-rabbit (Life Technologies, Carlsbad, CA, USA; 1:1000) was applied for 1h. Alexa-Fluor 488 phalloidin (Life Technologies, Carlsbad, CA, USA; 1:500) was added for 15min to selectively visualize F-actin. We used anti-neurofilament 200 monoclonal primary antibody (N0142, Sigma-Aldrich, Saint Louis, MO) and Alexa-Fluor 488 Goat anti-mouse as a secondary antibody to visualize spiral ganglions neurons. Whole-mount tissues were mounted in ProLong Gold Antifade Reagent (Life Technologies, Carlsbad, CA, USA). Confocal images were collected using Leica TCS SP5 confocal microscope (Leica Microsystems Inc., Bannockburn, IL, USA) and analyzed in LSM 5 Image Browser and Adobe Photoshop.

Transfected HEK293T and COS7 cells were fixed in 4% paraformaldehyde in 0.1 M PBS (pH 7.4); cells were permeabilized with 0.1% Trition-X100. Fixed cells were incubated with primary antibody at room temperature in PBS for 1.5hrs. The following primary antibodies were used: monoclonal Anti-FLAG (Sigma-Aldrich, St. Louis, MO, USA; 1:400) and Anti-cMYC (Sigma-Aldrich, St. Louis, MO, USA; 1:400). Secondary antibody incubation was for 1hr at room temperature. Secondary antibodies used: Alexa-Fluor-488 goat anti-mouse (Invitrogen, Grand Island, NY, USA; 1:500) to stain FLAG-tagged HOMER2 ^p.Arg185Pro^ and Alexa-Fluor-568 goat anti-rabbit (Invitrogen, Grand Island, NY, USA; 1:500) to stain cMYC-tagged HOMER2^WT^. F-actin was immuno-stained with Alexa-Fluor-647-phalloidin (Invitrogen, Grand Island, NY, USA;1:500). Cells were mounted in SlowFade Gold Antifade Reagent with DAPI (Life Technologies, Grand Island, NY, USA). Images were taken using a Zeiss LSM 510 with ZEN 2009 confocal microscope (Zeiss, Pleasanton, CA, USA).

### Plasmid constructs

The Gateway PLUS shuttle clone for HOMER2 (AF081530.1) was ordered from GeneCopoeia (GeneCopoeia Inc, Rockville, MD, USA). QuickChange Site-Directed Mutagenesis Kit (Stratagene, Cambridge, UK) was used for site-specific mutagenesis to introduce the P185 mutation (S4 Table). The mutant expression plasmid was sequence verified. Both full length open reading frames for WT protein HOMER2^WT^ and mutant HOMER2^p.Arg185Pro^ were cloned into the expression vectors pCMV-Tag3 (cMYC-tagged) and pCMV-Tag2 (FLAG-tagged), respectively (Agilent technologies, Santa Clara, CA, USA). All constructs were verified by sequence analysis.

### Cell culture

HEK293T cells and COS7 cells (ATCC, Manassas, VA, USA) were grown in Dulbecco's Modified Eagle's Medium (DMEM) supplemented with 10% FBS (Life Technologies, Carlsbad, CA, USA). Cells were incubated in a 5% CO2-humidified incubator at 37°C. Cells were grown on Poly-L-Lysine Coated coverslips (Corning, Tewksbury, MA, USA). Clonal cells were obtained by transfection with Transit-LT Transfection Reagent (Mirus Bio, Madison, WI USA) using cMYC-tagged HOMER2^WT^ and FLAG-tagged HOMER2^p.Arg185Pro^ plasmid constructs according to the manufacturer’s instructions.

### Morpholino knockdown and *in vivo* overexpression of mutant HOMER2

Zebrafish embryos were raised at 28.5°C as described [41]. All animal procedures were approved by the University of Iowa Office of Animal Resources (OAR) principle for the care and use of laboratory animals and the Institutional Animal Care and Use Committee (IACUC). Antisense morpholino oligonucleotides (MOs) were designed to block the exon/intron splice junctions between exon 1 and intron 1 (MO i1e1 5′- GGTACACATGTATCTGTCTGACCTT-3′) or intron 3 and exon 4 (5′-CGCAATGAAAACTGTAAACACTCTT-3′) of *homer2* (ENSDART00000124088) and bought from Gene Tools (Philomath, OR, USA). They were injected at 2.2 mg/ml along with 1 mg/ml p53 MO (5’-GCGCCATTGCTTTGCAAGAATTG-3’). A standard control MO (5’-CCTCTTACCTCAGTTACAATTTATA-3’) was used for injection of negative controls along with p53 MO. The efficacy of *homer2* knockdown by each morpholino was assessed by RT-PCR analysis with the following primer sets: forward (5’- GGTTCCCGCCAGTAAACAG-3’) and reverse (5’-GTTTGAGCTCCGTCTTCAGG-3’), which amplified the region between exons 1 and 12; B-actin primers were forward (5′-GAGATGATGCCCCTCGTG-3') and reverse 5'-GCTCAATGGGGTATTTGAGG-3'). MO i1e1 morpholino was used for all subsequent experiments. For i*n vivo* mRNA synthesis, HOMER2^WT^ and HOMER2^p.Arg185Pro^ plasmids were transferred into the expression vector pCS2+ with Gateway LR Clonase according to the manufacturer’s instructions (Life Technologies, Carlsbad, CA, USA). This cDNA was used as a template for HOMER2 capped mRNA synthesis using an Ambion mMESSAGE mMACHINE SP6 kit (Applied Biosystems, Foster City, CA, USA), and the product was tested for quality and yield by electrophoresis and spectroscopy (NanoDrop Thermo Scientific, Waltham, MA, USA) before injection. Microinjection was performed at the one- to two-cell stage using a microinjection system consisting of a SZX9 stereomicroscope (Olympus, Tokyo, Japan) and an IM300 Microinjector (Narishige, Tokyo, Japan). Overexpression of injected mRNA was assessed by quantitative PCR with the following primer sets: forward (5’-GACCCCAACACCAAGAAGAA-3’) and reverse (5’CACTGTGTTGGCTCTGCTGT-3’). Primers for B-actin were forward (5’-CGCGCAGGAGATGGGAACC-3’) and reverse (5’-CAACGGAAACGCTCATTGC-3’).

### Zebrafish hair cell staining

At 72 hours post fertilization (hpf), live larvae were submerged in 3 μM FM1-43 FX (Invitrogen, Grand Island, NY, USA) for 30 sec. They were then rinsed X3 in embryo media (ddH2O with 5.03 mM NaCl, 0.17 mM KCl, 0.33 mM CaCl2, 0.33 mM MgSO4, 0.1% w/v methylene blue) and fixed in 4% paraformaldehyde. Before viewing, fish were rinsed X3 in PBS and viewed in 75% glycerol, 25% PBS with a Zeiss 700 confocal microscope.

### Analysis of phenotypes in embryos and larvae

We focused on neuromasts that reside around the surface of the otic vesicle: o1, ml1, ml2, o2 and io4. Z-stacks were prepared using the max intensity z-projection function in ImageJ (NIH, Stapleton, New York City, USA). The morphous and structure of otic vesicles was observed in live larvae at 72hpf with a Leica MZFIII3 light microscope after anesthetizing with Tricaine. Images were analyzed with ImageJ (NIH).

### ABRs and DPOAEs

Mouse studies were carried out in accordance with University of Iowa Office of Animal Resources (OAR) principle for the care and use of laboratory animals and the Institutional Animal Care and Use Committee (IACUC). Mice were culled using methods approved by the American Veterinary Medical Association (AVMA) Guidelines for the Euthanasia of Animals.

The knockout *Homer2* ^*-/-*^ mice were donated by Paul F. Worley at John Hopkins University (Baltimore, Maryland, USA). These mice have a neomycin cassette inserted into exon 3 of *Homer2* abolishing gene expression [31]. The *Homer2* ^*-/-*^ colony was maintained on a C57BL/6J background. Mice were genotyped by PCR, as previously described (S8 Fig and S4 Table) [31]. Hearing thresholds were measured by click and tone burst (8, 16, and 32 kHz) ABR and DPOAE in *Homer2* ^*-/-*^, *Homer2* ^*+/-*^ and WT mice at two (P14), four (P28) and eight (P56) weeks. At least 23 animals were tested at each time point. Mice were anesthetized using intraperitoneal Ketamine/Xylazine at 0.1ml/10g body weight. Reference, ground and earth electrodes were placed subcutaneously just posterior to the tested ear (left ear), anterior to the contralateral ear and at the vertex of the head, respectively. ABRs were performed using an experimental setup and testing protocol, as described [42]. Briefly, clicks and tone-bursts were delivered to the testing ear through a plastic acoustic tube. ABRs were measured using an Etymotic Research ER10B+ probe microphone (Etymotic Research, Elk Grove, IL, USA) coupled to two Tucker-Davis Technologies MF1 multi-field magnetic speakers (Tucker-Davis Technologies, Alachua, FL, USA). Click and tone-burst stimuli were presented and recorded using custom software running on a PC connected to a 24-bit external sound card (Motu UltraLite mk3, Cambridge MA, USA). A custom-built differential amplifier with a gain of 1,000 dB amplified acoustic ABR responses. Output was passed through 6-pole Butterworth high-pass (100 Hz) and low-pass (3 kHz) filters and then to a 16-bit analog-to-digital converter (100,000 sample/s). Responses were recorded using standard signal-averaging techniques for 500 or 1000 sweeps. Hearing thresholds (db SPL) were determined by decreasing the sound intensity by 5 and/or 10 db decrements and recording the lowest sound intensity level resulting in a recognizable and reproducible ABR response wave pattern. Maximum ABR thresholds were capped at 100 db SPL. DPOAEs were measured unilaterally (left ear) using an experimental setup and testing protocol, as described [42]. In brief, DPOAE levels were elicited using two primary tone stimuli, f1 and f2, with sound pressure levels of 65 and 55 db SPL, respectively, with f2/f1 = 1.22. A custom plastic ear probe was inserted into the ear canal and DPOAE amplitudes were measured at f2 frequencies at 4000, 5657, 8000, 11314, 16000, 22627 and 32000 Hz and plotted after subtraction of noise floor amplitude.

### Ingenuity pathway analysis (IPA)

IPA (Ingenuity Systems, Mountain View, CA, USA) was used to map interactions between genes involved in NSHHL (86 genes) and HOMER2 and CDC42. Networks were created from user-specified seed molecules by searching the knowledge base for molecules that are known to biologically interact with the seeds and connecting them. Networks are displayed graphically as nodes (genes/gene products) and edges (the biological relationships between the nodes). IPA computes a score for each network according to the fit of all significant genes. A detailed description is given in the online repository (http://www.ingenuity.com).

### Statistical analysis

All results are displayed as mean ± standard error of the mean (mean ± SEM). Statistical analyses were performed using one-way ANOVA (Zebrafish data) or one-way ANOVA with post hoc T-test analysis using GraphPad Prism 6 (La Jolla, CA, USA) for ABR and DPOAE data. *P*-values < 0.05 were assigned as significant.

### Web resources

The URLs for data presented herein are as follows:

1000 Genomes; http://www.1000genomes.org

Hereditary Hearing Loss Homepage, http://hereditaryhearingloss.org

MutationTaster, http://www.mutationtaster.org

NHLBI Exome Sequencing Project Exome Variant Server; http://evs.gs.washington.edu/EVS/

Online Mendelian Inheritance in Man (OMIM), http://www.omim.org/

PolyPhen-2, http://genetics.bwh.harvard.edu/pph2/

RefSeq, http://www.ncbi.nlm.nih.gov/RefSeq

SIFT, http://sift.jcvi.org/

## Supporting Information

S1 Fig Audiological features. **(A)** Longitudinal binaural mean air conduction threshold data of affected family members. Age (years) is shown in symbol key. The panels are ordered (top left to bottom right) by age at last visit. **(B)** Cross-sectional linear regression analysis of binaural mean air conduction threshold on age (years) for each frequency separately. The regression line (dashed) is included for each frequency. Annual threshold deterioration (ATD, in bold print), that is the regression coefficient (dB/year), is included in each panel; asterisk indicates significant progression.
(TIF)

S2 Fig The p.Arg185Pro substitution does not alter subcellular localization of HOMER2. **(A-D)** Confocal microscopy images show expression of HOMER2 throughout the cytoplasm and co-localization of cMYC-tagged HOMER2^WT^ and FLAG-tagged HOMER2 ^p.Arg185Pro^ proteins expressed in HEK293T and COS7 cells. **(A)** Confocal images of cMYC-tagged HOMER2^WT^-transfected HEK293 cells immuno-stained with Alexa-Fluor-568 (red); F-actin immuno-stained with conjugated Alexa-Fluor-647-phalloidin (blue). **(B)** Confocal images of FLAG-tagged HOMER2 ^p.Arg185Pro^ -transfected HEK293 cells immuno-stained with Alexa-Fluor-488 (green); F-actin immuno-stained with conjugated Alexa-Fluor-647-phalloidin (blue). **(C)** Confocal images of HEK293 cells co-transfected with cMYC-tagged HOMER2^WT^ and FLAG-tagged HOMER2 ^p.Arg185Pro^ immuno-stained with Alexa-Fluor-568 (red) and Alexa-Fluor-488 (green), respectively; cell nuclei stained with DAPI (blue). **(D)** Confocal images of COS7 cells co-transfected with cMYC-tagged HOMER2^WT^ and FLAG-tagged HOMER2^p.Arg185Pro^ immuno-stained with Alexa-Fluor-568 and Alexa-Fluor-488, respectively; cell nuclei stained with DAPI (blue). The following primary antibodies were used: monoclonal Anti-FLAG and Anti-c-MYC antibodies (Sigma-Aldrich). Scale bar represents 10 μm.
(TIF)

S3 Fig Knock down of *homer2* in zebrafish embryos. **(A)** RT-PCR demonstrating knock down of *homer2* transcript using two morpholinos: MO e1i1 and MO i3e4. **(B)** Representative morphology of otic capsules in zebrafish embryos at 72 hpf either injected with a control MO or MO e1i1. **(C)** Otic capsule area was measured in 19 and 30 animals in the control and MO groups, respectively. There was no statistically significant difference between groups. Scale bar represents 50μm.
(TIF)

S4 Fig Overexpression of mutant HOMER2 in zebrafish embryos. Quantitative PCR assessing the relative quantity of mRNA from zebrafish embryos injected with WT HOMER2 (wtRNA) or HOMER2 P185-mutant RNA (P185RNA) demonstrates overexpression of both injected mRNAs.
(TIF)

S5 Fig Spiral ganglion morphology. **(A-D)** WT mice (n = 2). **(E-H)** *Homer2* ^*-/-*^ mice (n = 3). **(A and E)** Cochlea from 3 month old mice stained with DAPI (blue). **(B and F)** Spiral ganglions were labeled with anti-neurofilament antibody NF200 (green). **(C and G)** Merged pictures. **(D and H)** Gross morphology of the organ of corti labeled with DAPI and NF200 shows no obvious differences between WT and *Homer2* ^*-/-*^ mice. Scale bars in **A-C** and **E-G**: 50μm, **D** and **H**: 100μm.
(TIF)

S6 Fig HOMER2 interactome in the inner ear. Top-rated network generated by Ingenuity Pathway Analysis (IPA) (Ingenuity Systems www.ingenuity.com) using 89 known NSHL- causing genes (grey shade) and HOMER2 and CDC42 as seeds. A primary network (score 53) containing 28 of the seed proteins was generated and shows multiple interactions between molecules. HOMER2 interacts directly with CDC42 and F-Actin. CDC42 interacts either directly or indirectly with many proteins involved in NSHL (grey shade). Protein-protein interactions are indicated by lines and arrows. A detailed legend for the network shapes and molecular relationships is found in the IPA network (http://ingenuity.force.com/ipa/articles/Feature_Description/Legend).
(TIF)

S7 Fig HOMER2 antibody specificity validation. **(A)** Staining with F-actin shows three rows of OHCs and one row of IHCs in the cochlea. **(B)** Absence of Homer2 staining in the cochlea of *Homer2* ^*-/-*^ mice. **(C)** Merged pictures. Scale bar represents 10μm.
(TIF)

S8 Fig Genotyping of *Homer2* ^*-/-*^, *Homer2* ^*+/-*^ and *Homer2* ^*+/+*^ mice by PCR. PCR products were resolved on 2% agarose gel. The mutant allele corresponds to a 336-bp band. The wild type allele corresponds to a 237-bp band.
(TIF)

S1 Table WES and OtoSCOPE coverage statistics. (DOCX)

S2 Table Candidate variant list. (DOCX)

S3 Table Pathogenicity prediction for p.Arg185Pro mutation in HOMER2. (DOCX)

S4 Table List of primers and their applications. (DOCX)
