# Supplementary figures and images for: HOMER2, a Stereociliary Scaffolding Protein, Is Essential for Normal Hearing in Humans and Mice
Source: PLoS Genet. 2015 Mar 27;11(3):e1005137. doi: 10.1371/journal.pgen.1005137 (PMC4376867; doi:10.1371/journal.pgen.1005137)

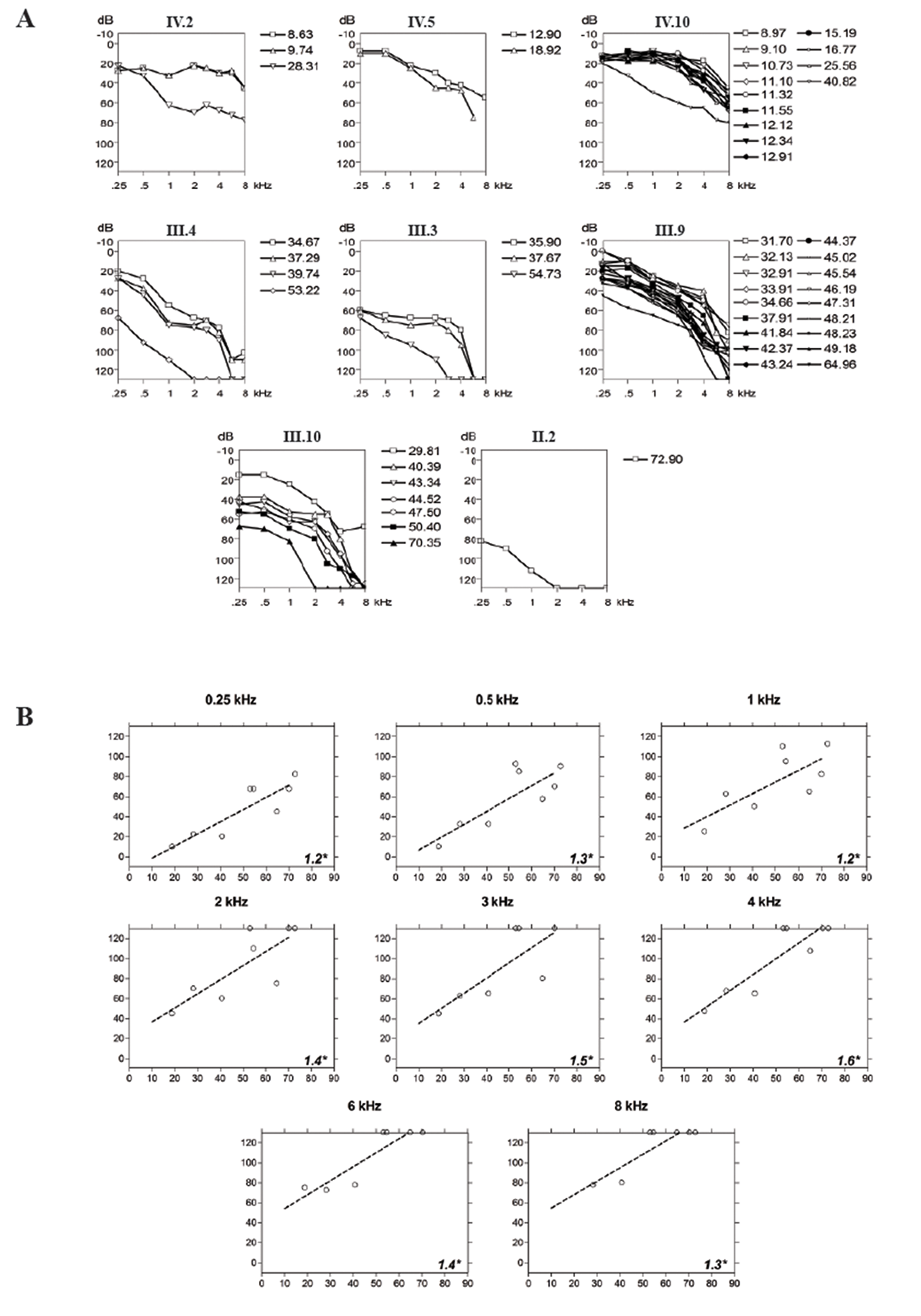

Supplement: S1 Fig — (A) Longitudinal binaural mean air conduction threshold data of affected family members. Age (years) is shown in symbol key. The panels are ordered (top left to bottom right) by age at last visit. (B) Cross-sectional linear regression analysis of binaural mean air conduction threshold on age (years) for each frequency separately. The regression line (dashed) is included for each frequency. Annual threshold deterioration (ATD, in bold print), that is the regression coefficient (dB/year), is included in each panel; asterisk indicates significant progression. (TIF) [file pgen.1005137.s001.tif]

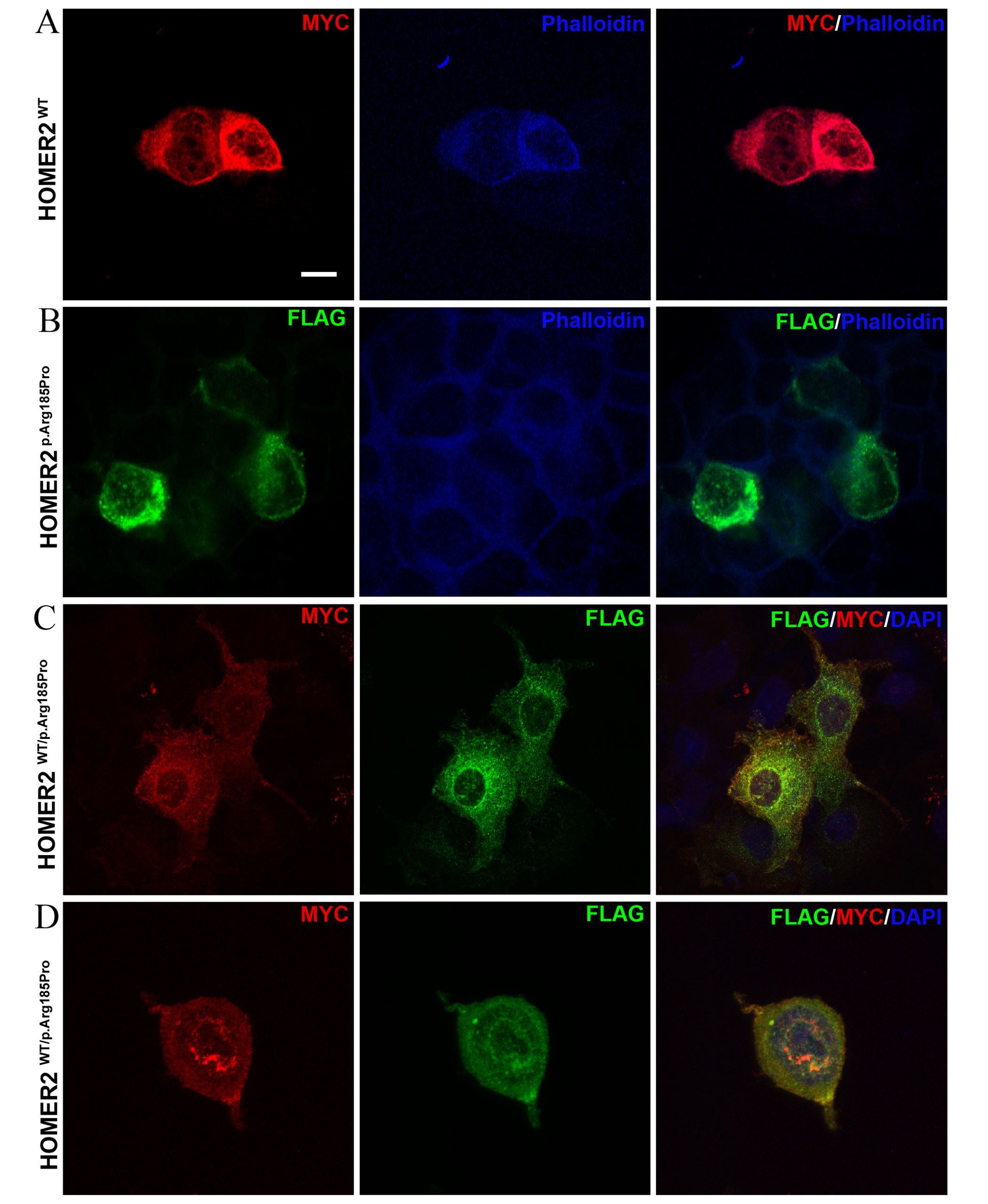

Supplement: S2 Fig — (A-D) Confocal microscopy images show expression of HOMER2 throughout the cytoplasm and co-localization of cMYC-tagged HOMER2WT and FLAG-tagged HOMER2 p.Arg185Pro proteins expressed in HEK293T and COS7 cells. (A) Confocal images of cMYC-tagged HOMER2WT-transfected HEK293 cells immuno-stained with Alexa-Fluor-568 (red); F-actin immuno-stained with conjugated Alexa-Fluor-647-phalloidin (blue). (B) Confocal images of FLAG-tagged HOMER2 p.Arg185Pro -transfected HEK293 cells immuno-stained with Alexa-Fluor-488 (green); F-actin immuno-stained with conjugated Alexa-Fluor-647-phalloidin (blue). (C) Confocal images of HEK293 cells co-transfected with cMYC-tagged HOMER2WT and FLAG-tagged HOMER2 p.Arg185Pro immuno-stained with Alexa-Fluor-568 (red) and Alexa-Fluor-488 (green), respectively; cell nuclei stained with DAPI (blue). (D) Confocal images of COS7 cells co-transfected with cMYC-tagged HOMER2WT and FLAG-tagged HOMER2p.Arg185Pro immuno-stained with Alexa-Fluor-568 and Alexa-Fluor-488, respectively; cell nuclei stained with DAPI (blue). The following primary antibodies were used: monoclonal Anti-FLAG and Anti-c-MYC antibodies (Sigma-Aldrich). Scale bar represents 10 μm. (TIF) [file pgen.1005137.s002.tif]

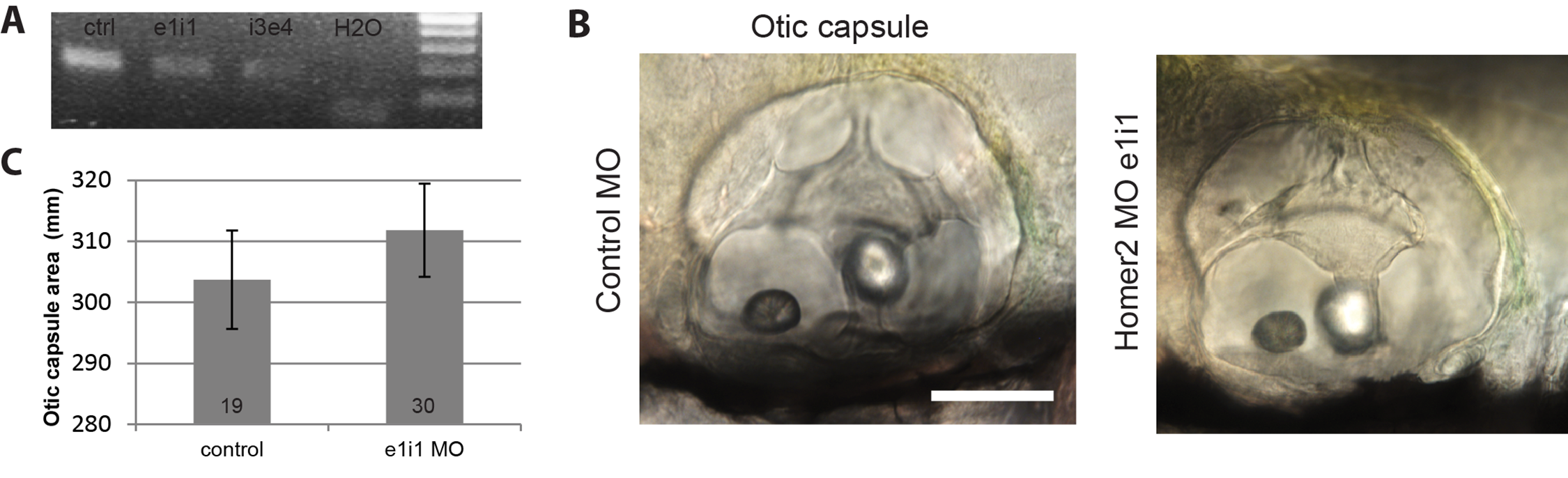

Supplement: S3 Fig — (A) RT-PCR demonstrating knock down of homer2 transcript using two morpholinos: MO e1i1 and MO i3e4. (B) Representative morphology of otic capsules in zebrafish embryos at 72 hpf either injected with a control MO or MO e1i1. (C) Otic capsule area was measured in 19 and 30 animals in the control and MO groups, respectively. There was no statistically significant difference between groups. Scale bar represents 50μm. (TIF) [file pgen.1005137.s003.tif]

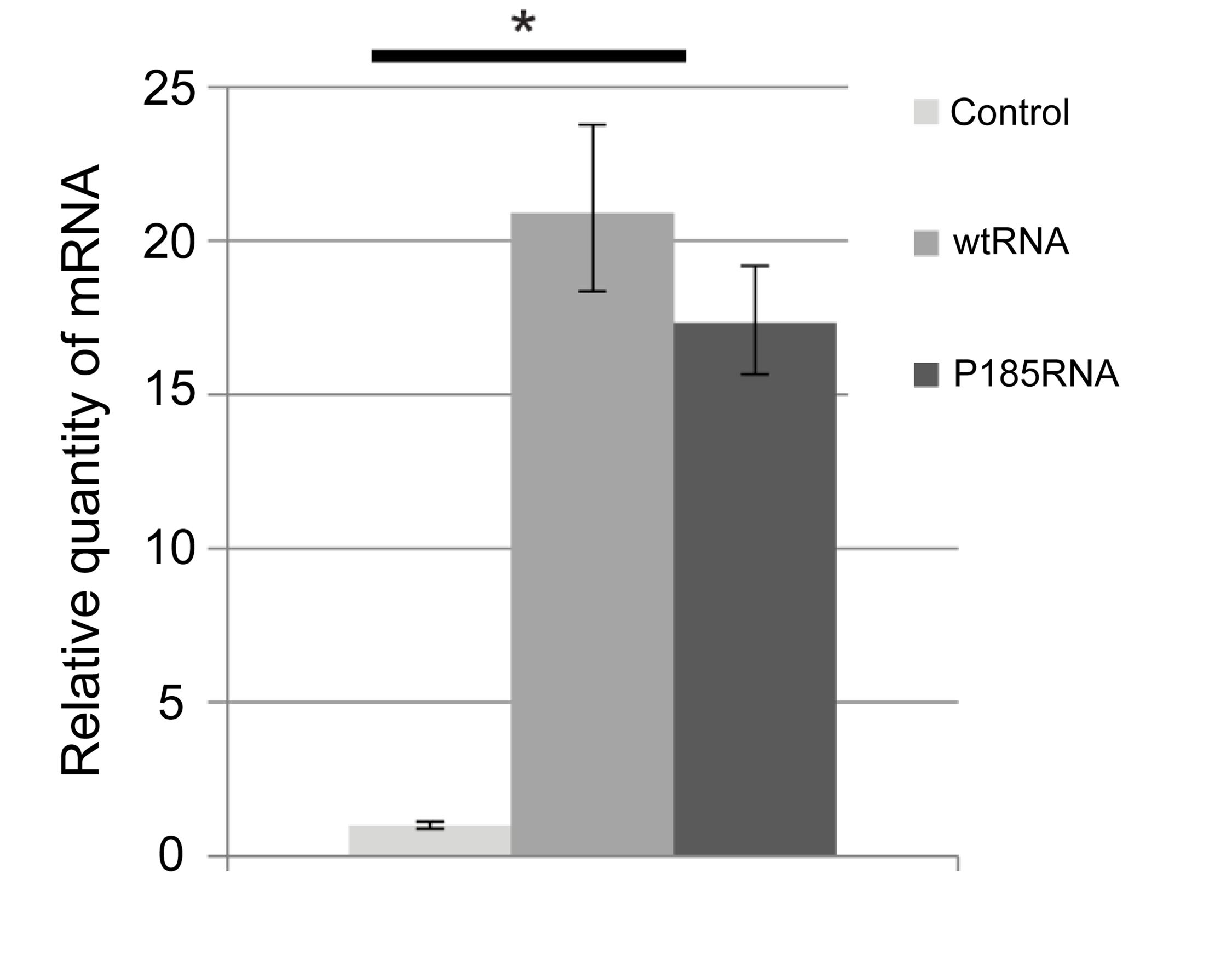

Supplement: S4 Fig — Quantitative PCR assessing the relative quantity of mRNA from zebrafish embryos injected with WT HOMER2 (wtRNA) or HOMER2 P185-mutant RNA (P185RNA) demonstrates overexpression of both injected mRNAs. (TIF) [file pgen.1005137.s004.tif]

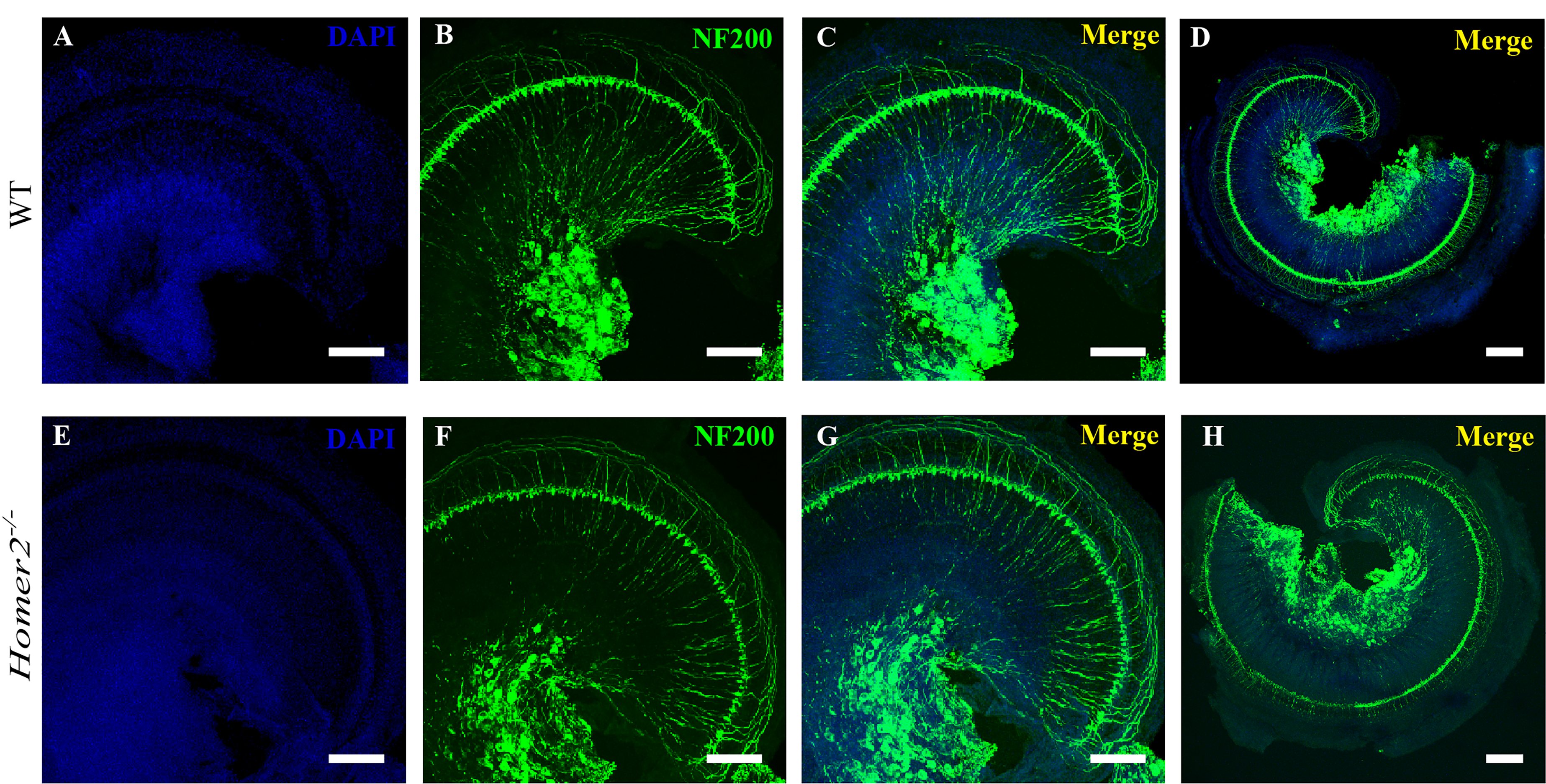

Supplement: S5 Fig — (A-D) WT mice (n = 2). (E-H) Homer2 -/- mice (n = 3). (A and E) Cochlea from 3 month old mice stained with DAPI (blue). (B and F) Spiral ganglions were labeled with anti-neurofilament antibody NF200 (green). (C and G) Merged pictures. (D and H) Gross morphology of the organ of corti labeled with DAPI and NF200 shows no obvious differences between WT and Homer2 -/- mice. Scale bars in A-C and E-G: 50μm, D and H: 100μm. (TIF) [file pgen.1005137.s005.tif]

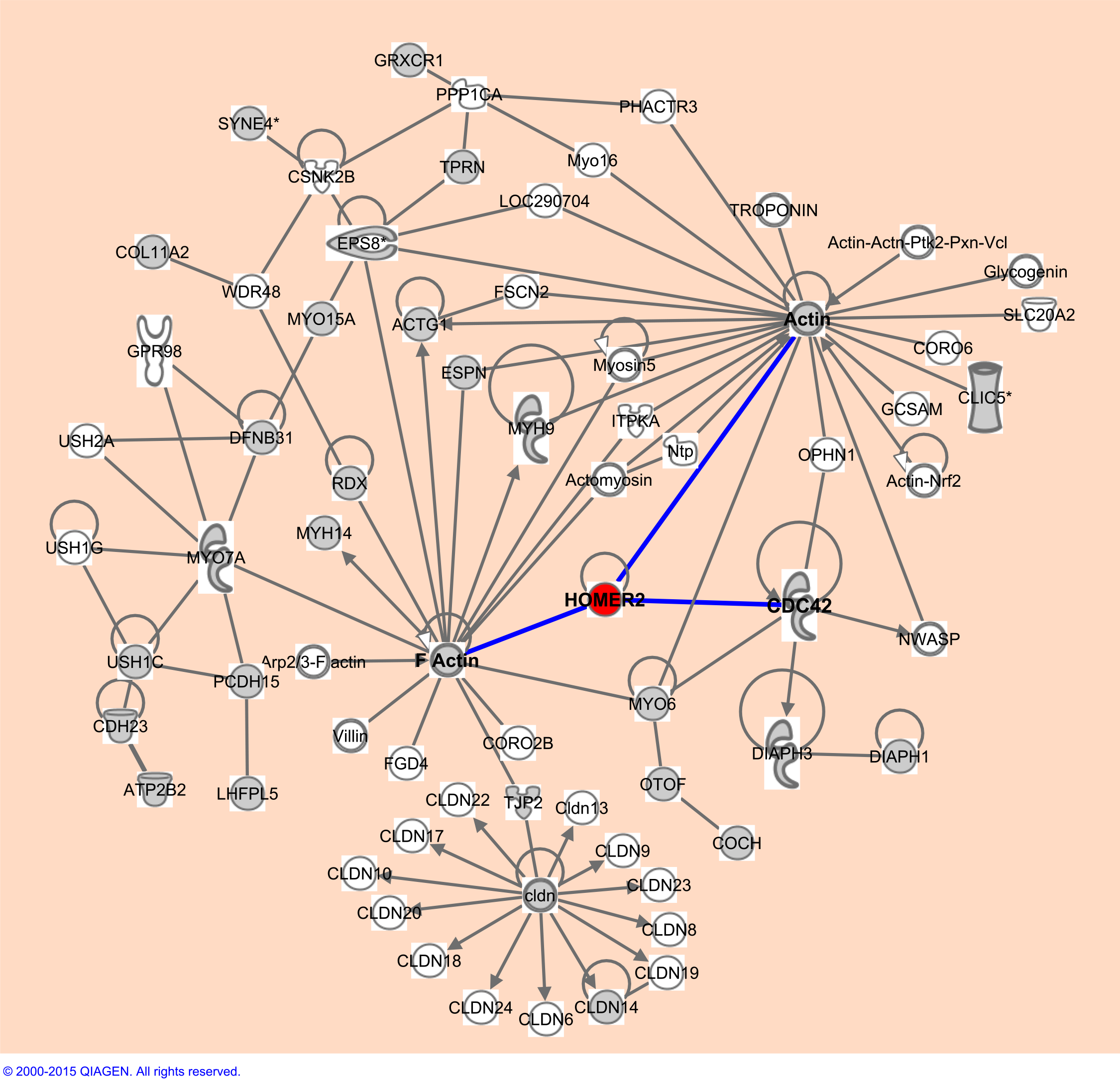

Supplement: S6 Fig — Top-rated network generated by Ingenuity Pathway Analysis (IPA) (Ingenuity Systems www.ingenuity.com) using 89 known NSHL- causing genes (grey shade) and HOMER2 and CDC42 as seeds. A primary network (score 53) containing 28 of the seed proteins was generated and shows multiple interactions between molecules. HOMER2 interacts directly with CDC42 and F-Actin. CDC42 interacts either directly or indirectly with many proteins involved in NSHL (grey shade). Protein-protein interactions are indicated by lines and arrows. A detailed legend for the network shapes and molecular relationships is found in the IPA network (http://ingenuity.force.com/ipa/articles/Feature_Description/Legend). (TIF) [file pgen.1005137.s006.tif]

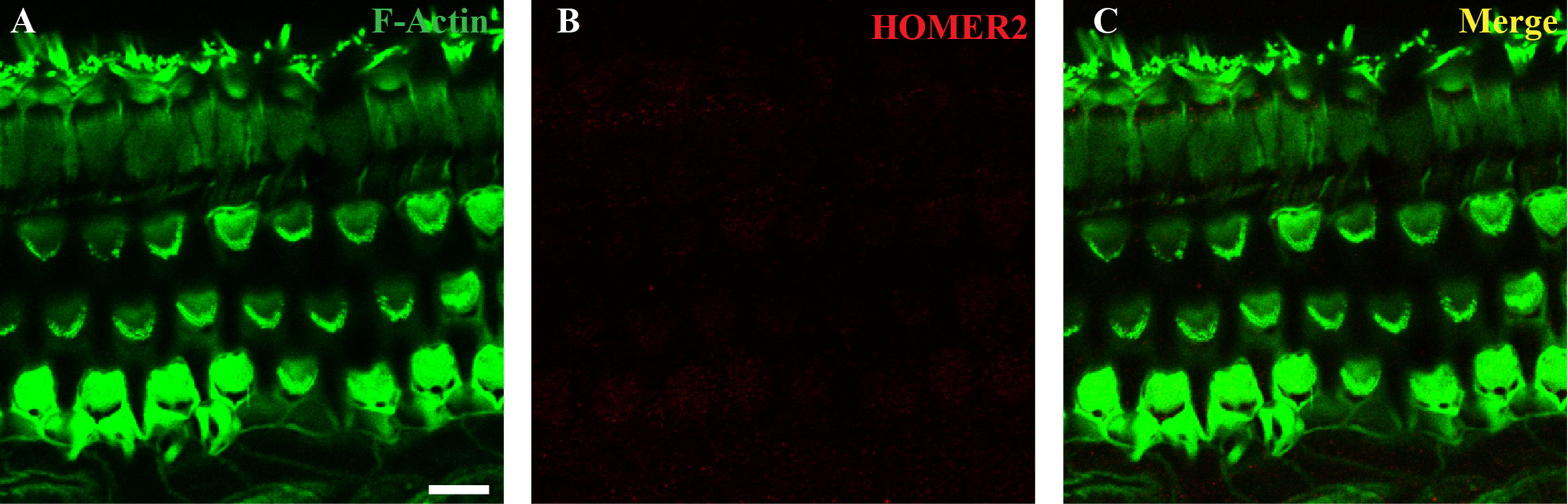

Supplement: S7 Fig — (A) Staining with F-actin shows three rows of OHCs and one row of IHCs in the cochlea. (B) Absence of Homer2 staining in the cochlea of Homer2 -/- mice. (C) Merged pictures. Scale bar represents 10μm. (TIF) [file pgen.1005137.s007.tif]

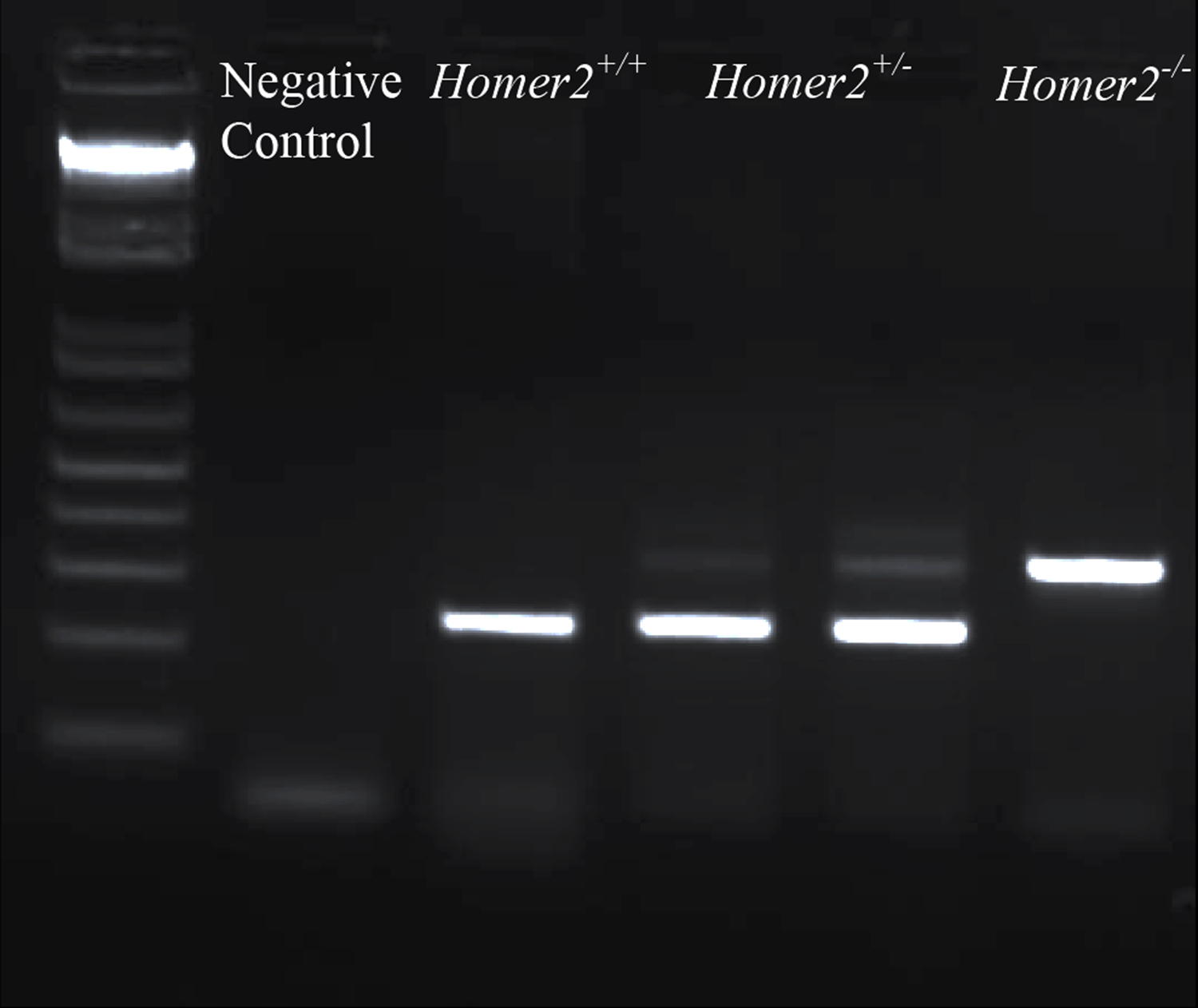

Supplement: S8 Fig — PCR products were resolved on 2% agarose gel. The mutant allele corresponds to a 336-bp band. The wild type allele corresponds to a 237-bp band. (TIF) [file pgen.1005137.s008.tif]
