# Supplementary material for: HOMER2, a Stereociliary Scaffolding Protein, Is Essential for Normal Hearing in Humans and Mice
Source: PLoS Genet. 2015 Mar 27;11(3):e1005137. doi: 10.1371/journal.pgen.1005137 (PMC4376867; doi:10.1371/journal.pgen.1005137)
Supplement: S1 Table — (DOCX) [file pgen.1005137.s009.docx]

**S1 Table: WES and OtoSCOPE coverage statistics**

|  | **WES** | | | | **OtoSCOPE** |
| --- | --- | --- | --- | --- | --- |
| **Sample** | **II.2** | **IV.5** | **IV.10** | **Average** |  |
| Target Coverage | 104.35 | 148.45 | 155.31 | 136.04 | 3518 |
| Number of Reads | 100223154 | 134412022 | 149684928 | 128106701 | 64385502 |
| % mapped reads | 97.25 | 97.78 | 96.99 | 97.34 | 52.80 |
| % Target Covered 1x | 99.43 | 99.42 | 97.8 | 98.88 | 99.9 |
| % Target Covered 10x | 95.07 | 96.62 | 91.68 | 94.46 | 99.7 |
| % Target Covered 20x | 90.38 | 93.33 | 87.48 | 90.40 | 99.6 |
| Total variants^a^ | 85990 | 82954 | 84553 | 84499 | 457 |
| putative pathogenic^b^ | 163 | 150 | 158 | 157 | 0 |

^a^passed quality filter

^b^heterozygous non-synonymous, splice site or indel variants with MAF <0.0005
