# Supplementary material for: HOMER2, a Stereociliary Scaffolding Protein, Is Essential for Normal Hearing in Humans and Mice
Source: PLoS Genet. 2015 Mar 27;11(3):e1005137. doi: 10.1371/journal.pgen.1005137 (PMC4376867; doi:10.1371/journal.pgen.1005137)
Supplement: S2 Table — (DOCX) [file pgen.1005137.s010.docx]

**S2 Table: Candidate variant list**

| **Gene** | **Variant** | **HGVS** | **MAF** | | |
| --- | --- | --- | --- | --- | --- |
|  |  |  | **1000G** | **EVS-AA** | **EVS-EA** |
| *BRPF1* | chr3:9785487:C>T | NM_001003694:c.2537C>T, p.Ser846Leu | unkn | unkn | unkn |
| *HOMER2* | chr15:83523493:C>G | NM_004839:c.554G>C, p.Arg185Pro | unkn | unkn | unkn |
| *LAMA2* | chr6:129581891:A>G | NM_000426:c.2132A>G, p.Tyr711Cys | unkn | 0.00% | 0.03% |
| *ZNF827* | chr4:146823734:T>C | NM_178835:c.677A>G, p.Lys226Arg | unkn | unkn | unkn |

EVS: Exome Variant Server; AA: African American; EA: European American

unkn: unknown
