# Supplementary material for: HOMER2, a Stereociliary Scaffolding Protein, Is Essential for Normal Hearing in Humans and Mice
Source: PLoS Genet. 2015 Mar 27;11(3):e1005137. doi: 10.1371/journal.pgen.1005137 (PMC4376867; doi:10.1371/journal.pgen.1005137)
Supplement: S3 Table — (DOCX) [file pgen.1005137.s011.docx]

**S3 Table: Pathogenicity prediction for p.Arg185Pro mutation in HOMER2**

| *In silico* Pathogenicity prediction tool | p.Arg185Pro prediction score |
| --- | --- |
| ^1^GERP++ | **Conserved (5.47)** |
| ^2^PhyloP | **Conserved (2.583)** |
| ^3^Polyphen2 HVAR | **Probably damaging (0.935)** |
| ^4^Polyphen2 HDIV | **Probably damaging (0.996)** |
| ^5^SIFT | **Damaging (0.01)** |
| ^6^LRT | **Deleterious** |
| ^7^Mutation Taster | **Disease causing (0.999)** |

^1^Scores < 0.0 (not conserved), > 0.0 (conserved)

^2^Scores < 0.95 (not conserved), ≥ 0.95 (conserved)

^3^Scores ranging from 0.0-0.446 (benign) 0.447-.909 (possibly damaging) 0.909-1 (probably damaging)

^4^Scores ranging from 0.0-0.446 (benign) 0.447-.909 (possibly damaging) 0.909-1 (probably damaging)

^5^Scores 0.00-0.05 = D (damaging) 0.06-1.0 = T (tolerated)

^6^Scores range from unknown, neutral, and deleterious

^7^Scores range from 0.0-0.5 (polymorphism) 0.5-1.0 (disease causing)
