# Supplementary material for: HOMER2, a Stereociliary Scaffolding Protein, Is Essential for Normal Hearing in Humans and Mice
Source: PLoS Genet. 2015 Mar 27;11(3):e1005137. doi: 10.1371/journal.pgen.1005137 (PMC4376867; doi:10.1371/journal.pgen.1005137)
Supplement: S4 Table — (DOCX) [file pgen.1005137.s012.docx]

**S4 Table: List of primers and their applications**

| Application | Sequence 5’-3’ |
| --- | --- |
| Sanger Sequencing of c.554G>C in *HOMER2*-6F | ATG-GGA-GAG-GCA-GCA-AGT-CT |
| Sanger Sequencing of c.554G>C in *HOMER*-6R | AGA-CCC-ACC-TGC-CAG-CTT-AC |
| Antisense MO oligonucleotides: MO i1e1 | GGT-ACA-CAT-GTA-TCT-GTC-TGA-CCT-T |
| Standard control MO | CCT-CTT-ACC-TCA-GTT-ACA-ATT-TAT-A |
| Site-Directed Mutagenesis/Forward | GGG-AGA-GCA-ATG-CAC-CGC-TGA-CCA-CAG-CAC-T |
| Site-Directed Mutagenesis/Reverse | AGT-GCT-GTG-GTC-AGC-GGT-GCA-TTG-CTC-TCC-C |
| Mouse Genotyping for Homer2 Wild Type Allele/Reverse | TAC-CTT-GGC-TCC-ATC-CAC-AC |
| Mouse Genotyping for Homer2 Mutant Allele/Reverse | GCC-ATA-ACA-AAA-GTC-CAG-TAT-GC |
| Mouse Genotyping for Homer2 Common/Forward | GGT-GGG-TGG-CCT-AGA-AAT-C |

MO: Morpholino
